# Intrapulmonary solitary fibrous tumor coexisting with lung adenocarcinomas

**DOI:** 10.1186/s40792-022-01508-4

**Published:** 2022-08-02

**Authors:** Shoei Kuroki, Takanori Ayabe, Toshihiro Gi, Yuichiro Sato, Hiroshi Nakada, Ryo Maeda

**Affiliations:** 1 Department of Thoracic and Breast Surgery, University of Miyazaki, Kihara 5200, Kiyotake, Miyazaki 889-1692 Japan; 2 Department of Pathology, University of Miyazaki, 5200 Kiyotake-cho kihara, Kiyotake, Miyazaki 889-1692 Japan; 3 Department of Diagnostic Pathology, University of Miyazaki, 5200 Kiyotake-cho kihara, Kiyotake, Miyazaki 889-1692 Japan; 4 Department of Radiology, University of Miyazaki, 5200 Kiyotake-cho kihara, Kiyotake, Miyazaki 889-1692 Japan

**Keywords:** Intrapulmonary solitary fibrous tumor, Surgery, Lung cancer

## Abstract

**Background:**

Solitary fibrous tumor (SFT) is a rare tumor of mesenchymal origin and accounts for < 2% of all soft tissue masses. Although SFT has been identified in multiple anatomic locations and can grow anywhere in the body, intrapulmonary SFT are rare.

**Case presentation:**

In this report, we presented a rare case of intrapulmonary solitary fibrous tumor (SFT) coexisting with lung adenocarcinoma in a 74-year-old man. Chest computed tomography showed a well-defined nodule with punctate calcification and measuring 2.3 × 2.1 cm and two ground-grass nodules with solid component. To obtain a definitive diagnosis and achieve complete resection, surgery was performed. The postoperative diagnosis was intrapulmonary SFT coexisting with lung adenocarcinoma. After surgery, he survived for 6 months without any signs of recurrence.

**Conclusion:**

Complete resection may be the best treatment for intrapulmonary SFT. Careful follow-up of the postoperative course is important, because differentiating between benignity and malignancy is difficult by histologic findings alone.

## Background

Solitary fibrous tumor (SFT) is a rare tumor of mesenchymal origin and accounts for < 2% of all soft tissue masses. Although SFT has been identified in multiple anatomic locations and can grow anywhere in the body, intrapulmonary SFT are rare. In this report, we described a case of resected intrapulmonary SFT coexisting with lung adenocarcinoma. The detailed clinical features of intrapulmonary SFTs remain unknown. In addition, we presented a review of intrapulmonary SFT cases (n = 51), including the present case, which have been reported in the English language literature.

## Case presentation

**Fig. 1:**
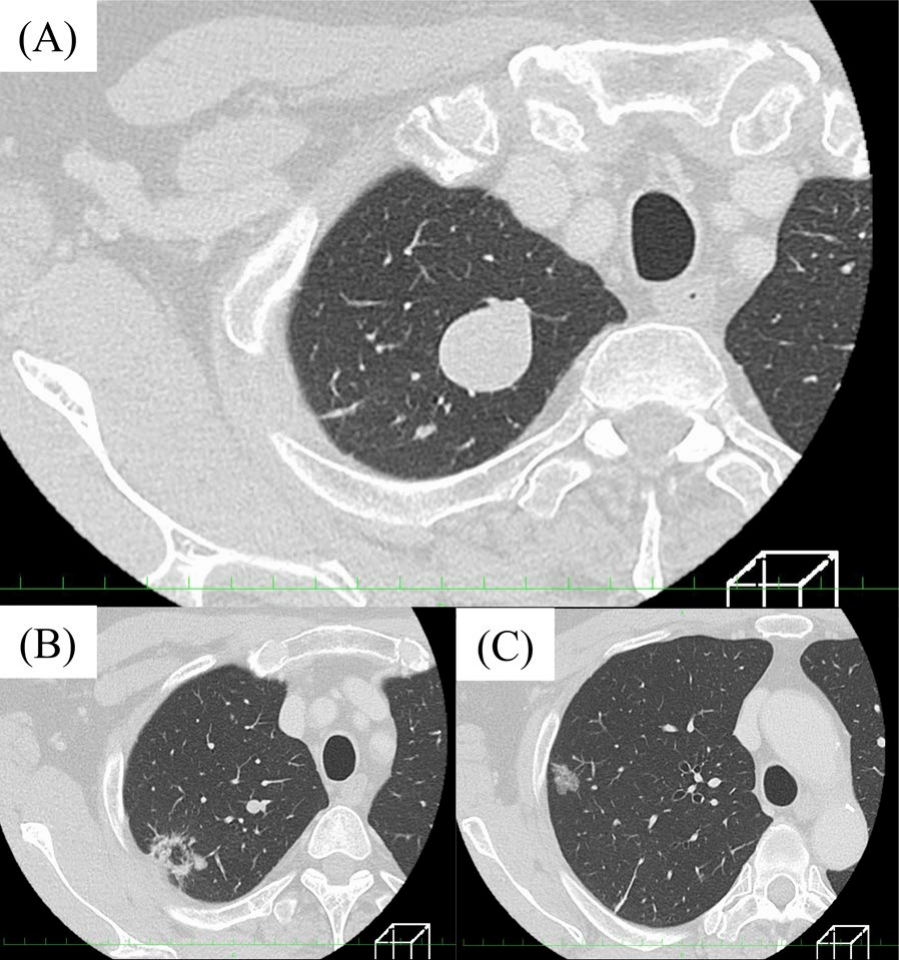
A computed tomographic scan of the chest shows showed a well-defined nodule with punctate calcification (**A**) and two ground-grass nodules with solid component (**B**)

A 74-year-old man who had prostate cancer was found to have abnormal shadows on computed tomography (CT) scan of the chest for metastatic workup. The chest CT scan showed a well-defined nodule with punctate calcification and measuring 2.3 × 2.1 cm and two ground-grass nodules with solid component (Fig. 1). Although the well-defined nodule did not change in size, the two ground-grass nodules slightly increased in size over 5 months. His clinical status was unremarkable. Hematologic examinations, including tests for tumor markers, such as carcinoembryonic antigen, cytokeratin fragment 21, and progastrin-releasing peptide, were within normal. Nevertheless, the two ground-grass nodules were highly suspicious for lung cancer. We performed right upper lobectomy and lymph node dissection in the hilum and mediastinum by video-assisted thoracic surgery.

**Fig. 2:**
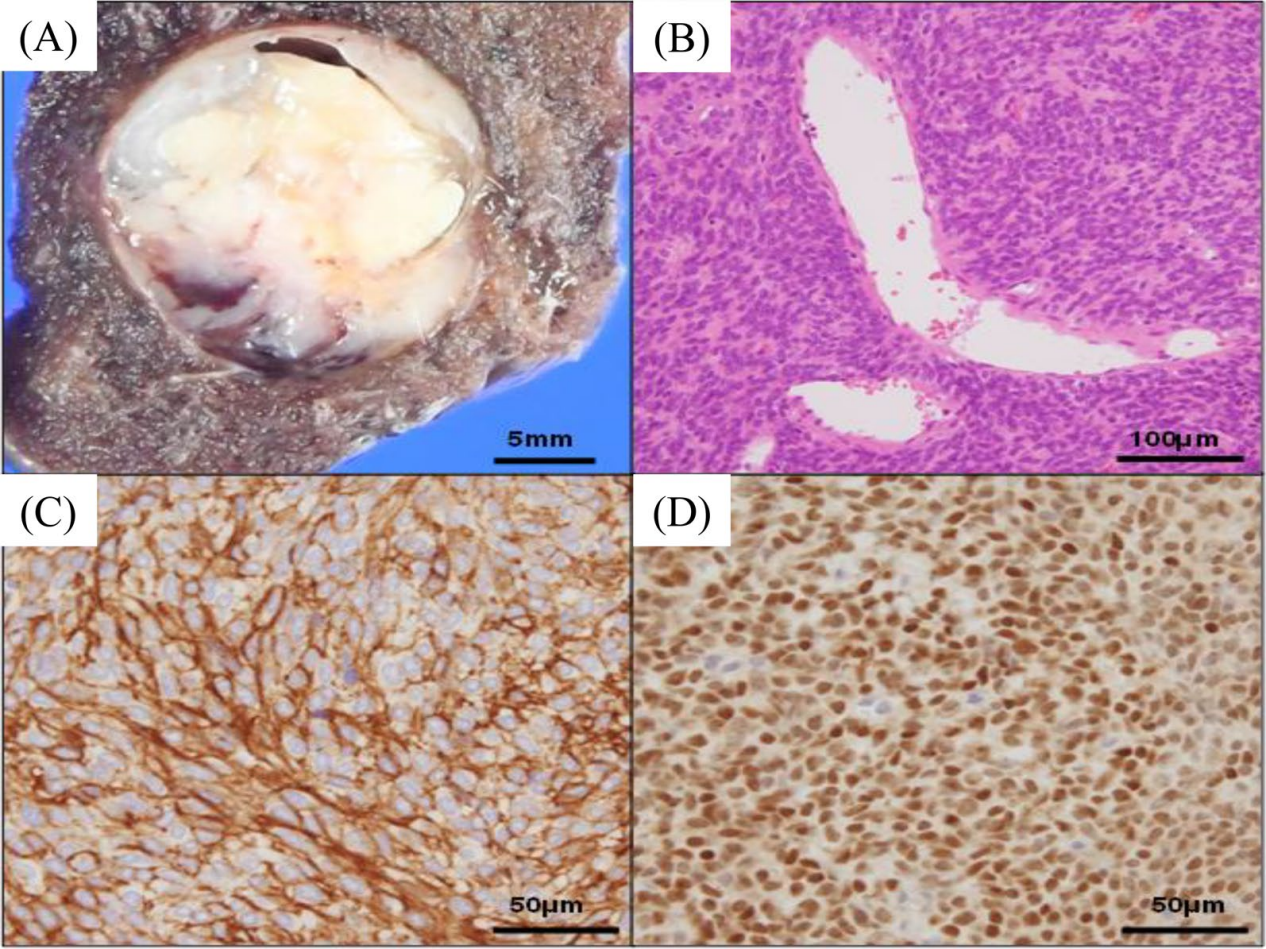
Macroscopical photograph shows a well-circumscribed tumor with white–yellow cut surface without involving the pleura (**A**). Microscopically, short spindled to oval cells proliferate in pattern-less pattern with branching vessels and collagenous stroma (**B**, hematoxylin and eosin stain). Immunohistochemically, the spindled tumor cells are diffusely and strongly positive for CD34 (**C**) and STAT6 (**D**)

**Fig. 3:**
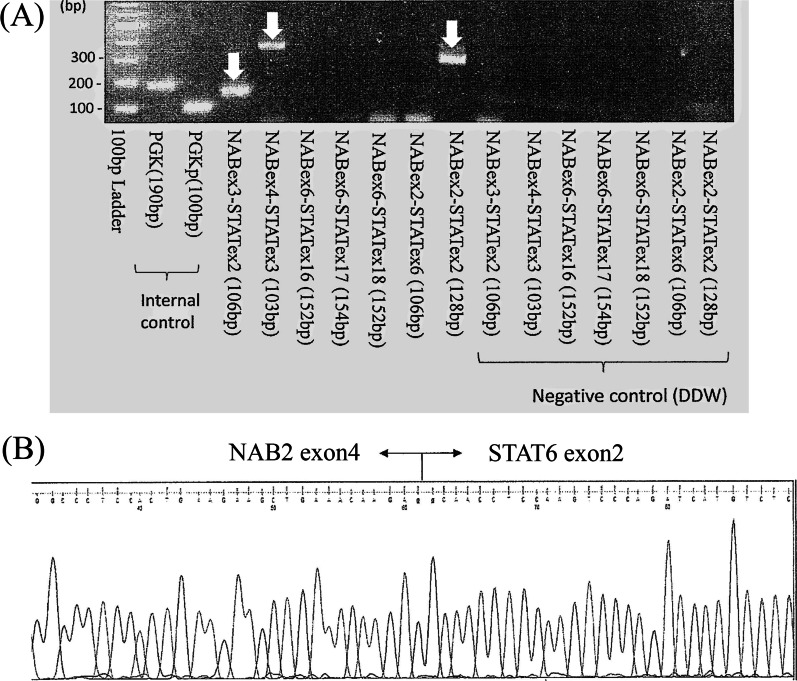
The NAB2 and STAT6 gene fusions (white arrows) are detected by reverse transcription-polymerase chain reaction (**A**). Direct sequence shows the NAB2 exon4-STAT6 exon2 gene fusion (**B**)

Histopathological examination revealed two lesions of lung adenocarcinoma were identified, and no lymph node metastasis was seen. The well-circumscribed tumor was composed of short spindled to oval cells without involving the pleura (Fig. 2, B). These spindled cells proliferate in pattern-less pattern or sheeted pattern with branching vessels, collagen stroma, and myxoid degeneration (Fig. 2B). There was no high-mitotic activity (0/10 high-power fields), tumor necrosis, or vessel permeation. Immunohistochemically, the spindle cells were diffusely positive for CD34 and STAT 6 and negative for cytokeratin (Fig. 2C, d). The MIB-1 index of the short spindle cells was 7.6%. Additionally, the NAB2 and STAT6 gene fusions were detected by reverse transcription-polymerase chain reaction (Fig. 3A) and direct sequence (Fig. 3B). The final diagnosis was intrapulmonary SFT coexisting with lung adenocarcinoma with no lymph node metastases. He survived for 6 months after surgery without any signs of recurrence.

## Discussion

SFT was first described in 1931 by Klemperer and Rabin as a distinct mesothelial tumor arising from the pleura [1]. Thereafter, several synonyms, such as localized mesothelioma, fibrous localized mesothelioma, benign fibrous mesothelioma, and pleural fibroma, have been used because of its heterogenetic theory of origin. Currently, SFTs are widely recognized as mesenchymal neoplasms based on immunohistochemical and ultrastructural findings. In the 2020 World Health Organization (WHO) classification of soft tissue tumors, the terminology has been unified under SFT only [2]. Thus far, SFTs have been reported in other numerous sites, such as the peritoneum, parotid gland, paranasal sinuses, orbit, skin, and intracranial areas [3], thereby, supporting a mesenchymal rather than a mesothelial origin. Although SFT can occur at nearly all anatomic locations, intrapulmonary SFTs are relatively rare. The development of intrapulmonary SFT may be attributed to (i) the direct continuity between the subpleural mesenchyme and interlobular septa or (ii) the presence of lung fibroblasts in the submesothelial areas of normal pulmonary parenchyma [4].

Histologically, spindle-shaped cells may be seen in fibrosarcoma, leiomyosarcoma, schwannoma, and others. Therefore, the diagnosis of SFTs may not be confirmed without immunohistochemical staining. The most valuable immunohistochemical markers in the diagnosis of SFTs are CD34 and STAT6, which were diffusely and strongly positive in the present case. On a molecular level, SFTs have been shown to be pathogenetically linked to a gene fusion secondary to a paracentric inversion on chromosome 12q13 and involves NAB2 and STAT6 [5], which are highly sensitive and specific markers for SFT. In the present case, the *NAB2*-*STAT6* gene fusions detected by reverse transcription-polymerase chain reaction and direct sequence led to the definitive diagnosis of SFT. According to the WHO classification [2], the prediction of metastatic risk in SFTs are follows: (1) patient age in years (≥ 55); (2) mitoses per 10 high-power fields; (3) tumor size in cm, and (4) tumor necrosis. The tumor in the present case was considered as low risk of metastasis.

To date, 50 cases of intrapulmonary SFT have been reported in the English language literature [4, 6–25]. We summarized a total of 51 cases, including the present case, with a literature review of intrapulmonary SFT cases (Table 1). All patients, except one, were treated by surgical resection, which encompassed wedge resection to pneumonectomy. Histologically, five tumors showed intermediate or malignant features. Although multiple factors, such as age and tumor size, have been associated with survival [26–28], higher histologic grade has been considered to have the strongest correlation with prognosis. Of these five tumors diagnosed as intermediate or malignant SFT, two (40%) had recurrence.

**Table 1:** Characteristics of the intrapulmonary solitary fibrous tumors present in the English language literature

No. of studies (cases)	22 (51)
Age (years)	
Mean	58
Range	7–83
Sex	
Male	27
Female	24
Tumor sizes (mm)	
Mean	63
Range	12–220
Symptoms	
Unknown	24
Asymptomatic	19
Cough	3
Chest pain	3
Chest discomfort	1
Symptomatic hypoglycemia	1
Localization	
Unknown	7
Right lung	2
Left lung	5
Right upper lobe	8
Right middle lobe	2
Right lower lobe	4
Left upper lobe	4
Left lower lobe	19
Treatment	
Surgery	50
Radiofrequency ablation	1
Histology	
Benign	46
Intermediate or malignant	5

Resection with free margins is considered the treatment for intrapulmonary SFT. Adequate wedge resection, anatomic segmentectomy, and lobectomy, depending on the location of the mass, are the common procedures for surgical resection of intrapulmonary tumors. Outcomes have been favorable in a majority of patients histologically diagnosed as benign SFTs. However, tumor behavior does not always correlate with histologic findings, because a few histologically benign SFTs reportedly recurred [19, 21]. Rao et al. [19] reported metastases in 10% of benign cases of intrapulmonary SFT. Inoue et al. [21] documented local recurrence of SFT 2 years after complete wedge resection of the left upper lobe and emphasized the importance of longer-term postsurgical follow-up, even for benign-appearing tumors. Judging the tumor as benign or malignant solely by histologic findings is considered to be difficult. Therefore, we should be careful to follow the postoperative course of patients, even in cases histologically diagnosed as benign SFT.

## Conclusion

Complete resection may be the best treatment for intrapulmonary SFT. Careful follow-up of the postoperative course is important, because differentiating between benignity and malignancy is difficult by histologic findings alone.

## Abbreviations

SFT: Solitary fibrous tumor
CT: Computed tomography
WHO: World Health Organization

## References

[1] Klemperer P, Coleman BR. Primary neoplasms of the pleura: A report of five cases. Am J Ind Med. 1992; 22: 1–31. 10.1002/ajim.4700220103 1415270

[2] WHO Classification of Tumours Editorial Board. Soft tissue and bone tumours. Lyon: International Agency for Research on Cancer; 2020.

[3] Zhang K, Liu HJ, Cheng ZB, Deng M, Luo J, Qi X (2020). Solitary fibrous tumor: A 10-year retrospective analysis with several rare cases. Chin Med J (Engl).

[4] Kouki HS, Koletsis EN, Zolota V, Prokakis C, Apostolakis E, Dougenis D (2008). Solitary fibrous tumor of the lung. Gen Thorac Cardiovasc Surg.

[5] Chmielecki J, Crago AM, Rosenberg M, O’Connor R, Walker SR, Ambrogio L (2013). Whole-exome sequencing identifies a recurrent NAB2-STAT6 fusion in solitary fibrous tumors. Nat Genet.

[6] Caruso RA, LaSpada F, Gaeta M, Minutoli I, Inferrera C (1996). Report of an intrapulmonary solitary fibrous tumor: Fine-needle aspiration cytologic findings, clinicopathological, and immunohistochemical features. Diagn Cytopathol.

[7] Khalifa MA, Montgomery EA, Azumi N, Gomes MN, Zeman RK, Min KW (1997). Solitary fibrous tumors: A series of lesions, some in unusual sites. South Med J.

[8] Sironi M, Rho B, Spinelli M (2005). Adenofibromatous pattern in a solitary fibrous tumor of the lung. Int J Surg Pathol.

[9] Kanamori Y, Hashizume K, Sugiyama M, Motoi T, Fukayama M, Ida K (2005). Intrapulmonary solitary fibrous tumor in an eight-year-old male. Pediatr Pulmonol.

[10] Patsios D, Hwang DM, Chung TB (2006). Intraparenchymal solitary fibrous tumor of the lung: An uncommon cause of a pulmonary nodule. J Thorac Imaging.

[11] Fridlington J, Weaver J, Kelly B, Kelly E (2007). Secondary hypertrophic osteoarthropathy associated with solitary fibrous tumor of the lung. J Am Acad Dermatol.

[12] Baliga M, Flowers R, Heard K, Siddiqi A, Akhtar I (2007). Solitary fibrous tumor of the lung: A case report with a study of the aspiration biopsy, histopathology, immunohistochemistry, and autopsy findings. Diagn Cytopathol.

[13] Sagawa M, Ueda Y, Matsubara F, Sakuma H, Yoshimitsu Y, Aikawa H (2007). Intrapulmonary solitary fibrous tumor diagnosed by immunohistochemical and genetic approaches: Report of a case. Surg Today.

[14] Sakurai H, Tanaka W, Kaji M, Yamazaki K, Suemasu K (2008). Intrapulmonary localized fibrous tumor of the lung: A very unusual presentation. Ann Thorac Surg.

[15] Cardinale L, Ardissone F, Cataldi A, Familiari U, Solitro F, Fava C (2009). Solitary fibrous tumor of the lung: Three rare cases of intraparenchymal nodules. Acta Radiol.

[16] Geramizadeh B, Banani A, Moradi A, Hosseini SM, Foroutan H (2010). Intrapulmonary solitary fibrous tumor with bronchial involvement: A rare case report in a child. J Pediatr Surg.

[17] Kawaguchi K, Taniguchi T, Usami N, Yokoi K (2011). Intrapulmonary solitary fibrous tumor. Gen Thorac Cardiovasc Surg.

[18] Barrettara B, Napoli G, Lacitignola A, Sardelli P (2013). Fibrous lung tumor: A peculiar case. J Thorac Dis.

[19] Rao N, Colby TV, Falconieri G, Cohen H, Moran CA, Suster S (2013). Intrapulmonary solitary fibrous tumors: Clinicopathologic and immunohistochemical study of 24 cases. Am J Surg Pathol.

[20] Dong A, Zuo C, Wang Y, Cui Y (2014). Enhanced CT and FDG PET/CT in malignant solitary fibrous tumor of the lung. Clin Nucl Med.

[21] Inoue T, Owada Y, Watanabe Y, Muto S, Okabe N, Yonechi A (2016). Recurrent intrapulmonary solitary fibrous tumor with malignant transformation. Ann Thorac Surg.

[22] Arsalane A, Zidane A, Fenane H, Azami A, Essadi I, Raissi A, et al. Solitary fibrous tumor: Case report of intrapulmonary location. Case Rep Oncol Med. 2018; 556:5745471. 10.1155/2018/5745471 PMC6304895 30631619

[23] Lin X, Xiang Y, Shi H, Zhang F (2018). Primary intrapulmonary solitary fibrous tumours. Oncol Lett.

[24] Alghamdi ZM, Othman SA, Al-Yousef MJ, AlFadel BZ (2020). Intrapulmonary location of benign solitary fibrous tumor. Ann Thorac Med.

[25] van Leeuwen RJH, Brunner S, Pojda J, Diebold J, Kestenholz P, Minervini F (2021). Intrapulmonary solitary fibrous tumor with adenofibromatous pattern with features of pleomorphic high grade sarcoma-a case report and an overview of the differential diagnosis. Quant Imaging Med Surg.

[26] Gold JS, Antonescu CR, Hajdu C, Ferrone CR, Hussain M, Lewis JJ (2002). Clinicopathologic correlates of solitary fibrous tumors. Cancer.

[27] Demicco EG, Park MS, Araujo DM, Fox PS, Bassett RL, Pollock RE (2012). Solitary fibrous tumor: A clinicopathological study of 110 cases and proposed risk assessment model. Mod Pathol.

[28] England DM, Hochholzer L, McCarthy MJ. Localized benign and malignant fibrous tumors of the pleura. A clinicopathologic review of 223 cases. Am J Surg Pathol. 1989; 13: 640–658. 10.1097/00000478-198908000-00003 2665534

